# Cystoscopic‐Guided Laser Ablation of Intramural Ectopic Ureters in Male Dogs: A Retrospective Study of 18 Cases (2011–2023)

**DOI:** 10.1111/jvim.70243

**Published:** 2025-09-10

**Authors:** Josh S. Chang, Allyson C. Berent, Chick W. Weisse

**Affiliations:** ^1^ Schwarzman Animal Medical Center New York New York USA

**Keywords:** dilated, incontinence, urethra, urinary

## Abstract

**Background:**

There are limited studies on cystoscopic‐guided laser ablation for treating ectopic ureters in male dogs. Further investigation is needed to assess its safety and efficacy.

**Hypothesis/Objective:**

Retrospectively describe long‐term outcomes in male dogs treated using cystoscopic‐guided laser ablation of ectopic ureters (CLA‐EU).

**Animals:**

Eighteen client‐owned male dogs.

**Methods:**

Medical records of male dogs that had CLA‐EU performed were retrospectively reviewed. Continence scores were assigned before and after the procedure. Data collected included signalment, neuter status, age of onset of urinary incontinence, pre‐ and post‐operative continence scores, CBC, serum biochemistry, urinalysis, microbiological urine culture, pre‐ and post‐operative abdominal imaging, EU laterality, concurrent anatomic anomalies, laser type and size used, procedure time, complications, and follow‐up.

**Results:**

The procedure was performed successfully in all 28 ureters in the 18 dogs. Post‐operative continence scores were significantly improved from pre‐operative scores (*p* = 0.001). Four (22%) dogs were continent at the time of diagnosis. Of the 14 dogs initially incontinent, 11/14 (79%) became continent after CLA‐EU without additional treatments, and an additional dog became continent with the addition of medications. Preoperatively, 14/28 kidneys and 17/28 ureters had evidence of hydronephrosis and hydroureter, respectively. Of the dogs, 10/14 with hydronephrosis and 12/17 with hydroureter showed improvement on post‐operative ultrasonography. The median follow‐up time was 1789 days (range, 98–3560).

**Conclusion and Clinical Importance:**

In male dogs, CLA‐EU was a safe, effective, and minimally invasive procedure with good to excellent short‐ and long‐term outcomes, achieving continence in most dogs and improvement or stabilization of associated hydronephrosis and hydroureter.

**Abbreviations**
ACVIM: American College of Veterinary Internal Medicine
BUN: blood urea nitrogen
CLA: cystoscopic‐guided laser ablation
CLA‐EU: cystoscopic‐guided laser ablation of ectopic ureter
EU: ectopic ureter
PPA: phenylpropanolamine
USG: urine specific gravity
UTI: urinary tract infection

## 1 Introduction

Ectopic Ureters (EU) are congenital malformations characterized by abnormal termination of one or both ureteral orifices along the urogenital tract distal to the trigone of the urinary bladder. Ectopic ureters are further categorized as extramural or intramural, with extramural EUs bypassing the urinary bladder completely and implanting distal to the urinary bladder [1]. Intramural EUs make up > 95% of EU cases in dogs and are characterized by normal extramural insertion at the level of the bladder neck, with submucosal tunneling caudally and subsequent opening of the orifice in the urogenital tract distal to the urinary bladder [2, 3]. Ectopic ureters are reported in many breeds, but more frequently in the Labrador Retriever, Golden Retriever, Siberian Husky, West Highland White Terrier, Wire Haired Fox Terrier, Newfoundland, and Poodle [1, 4, 5, 6, 7, 8, 9].

Cystoscopic‐guided laser ablation of ectopic ureters (CLA‐EU) has been reported to be safe and effective in treating female dogs with intramural EUs over the past two decades [7, 10, 11, 12, 13]. Despite achieving substantial success with the use of these minimally invasive procedures in female dogs with intramural EUs, studies evaluating the efficacy of such procedures in a large cohort of male dogs are not available. One case series in the veterinary literature reported the success of CLA‐EU in four male dogs with intramural EUs [6]. Positive outcomes were reported, with all dogs being continent with a median follow‐up period of 18 months without the need for additional interventions. Additional studies in larger cohorts of male dogs using CLA‐EU or in a cohort of dogs using the newly described technique of percutaneous perineal access for cystourethroscopy have not been reported since 2008 [6, 14].

Our objective was to retrospectively describe short‐ and long‐term outcomes in a cohort of male dogs using CLA‐EU to correct intramural EUs via perineal access.

## 2 Materials and Methods

A retrospective review of medical records of dogs was performed to identify male dogs diagnosed with intramural ectopic ureters between January 2011 and December 2023 that had CLA‐EU performed at the Schwarzman Animal Medical Center. Inclusion criteria were a definitive diagnosis of EU confirmed using retrograde flexible cystourethroscopy and follow‐up time of > 90 days. No additional procedures or treatments were administered during the study period other than those prescribed by the authors. Data collected included signalment, neuter status, age of onset of urinary incontinence, pre‐operative continence score (Table S1), pre‐operative CBC, serum biochemistry, urinalysis, urine microbiological culture, pre‐operative abdominal imaging findings, laterality and location of EUs, concurrent urinary tract anatomic anomalies, technique and laser type and size used, procedure time, post‐procedure continence score, post‐procedure imaging findings, post‐procedure laboratory findings, complications, and follow‐up time.

Owners were contacted either by phone or email post‐operatively to obtain follow‐up data including neuter status of previously intact dogs, if additional medical or surgical interventions were performed elsewhere, post‐operative abdominal ultrasonographic findings, and urinary continence scores (Table 1). The urinary continence score system was modified based on continence scales used in previous studies [7, 15].

**TABLE 1:** Modified urinary continence scoring system [7, 15].

Urinary continence score	Description
1	Leaking always and does not accumulate urine in the bladder at rest. Fully incontinent
2.5	Leaking at all times: running, playing, or laying down. Can urinate a stream of urine and has urine accumulating in the bladder at rest
5	Leaking mainly when laying down, minimally when walking or playing, may leak immediately before or after urination
7.5	Only leaking when resting and laying down
9	No puddles of urine noted. Urine only detected to leak on fur/around prepuce few times a week
10	No leaking at all. Fully continent

Each dog had retrograde flexible cystourethroscopy performed in dorsal recumbency for confirmation of the EU(s) before obtaining perineal access for rigid cystoscopy, as described previously [14]. Once access was achieved, a 2.7 mm 30‐degree rigid cystoscope was used to identify each EU. A combination of a 0.025″ angle‐tipped hydrophilic guidewire with a 4 Fr open‐ended ureteral catheter was advanced into the EU orifice under cystoscopic and fluoroscopic guidance. A retrograde ureteropyelogram and cystourethrogram were performed using iohexol (Omnipaque, GE Healthcare) to confirm an intramural EU. The presence of hydroureter, hydronephrosis, narrowed ureteral opening, ureterocele, dilated urethral lumen, or some combination of these was documented when present. The cystoscope then was removed over the guidewire and catheter combination, leaving the catheter in the ureter, and inserted back into the urethral sheath, next to the catheter. A 400‐ or 600‐μm laser fiber was inserted through the working channel of the cystoscope using either a diode laser set at 5–8 W or a Holmium:YAG laser set at 0.9–1.2 J and 9–12 Hz. Next, the intramural portion of the EU was ablated, extending from the EU orifice within the urethral lumen into the urinary bladder, stopping approximately 1 cm cranial to the urethrovesicular junction before the point where the ureter was documented by fluoroscopy to traverse from an intra‐ to extramural location. Any concurrent ureterocele also was ablated at the same time. For dogs with bilateral EUs, the procedure was repeated on the contralateral side, generally starting with the more proximal EU and finishing with the more distal EU.

## 3 Statistical Analysis

Statistical analyses were performed using SAS Version 9.4 (SAS Institute Inc., Cary, NC). The Wilcoxon rank sum test was used to compare continence scores before and after the procedure as ordinal data. Variables including age at presentation, weight, neuter status, pre‐operative laboratory test results (blood urea nitrogen [BUN], symmetric dimethylarginine [SDMA], creatinine, urine specific gravity, presence of urinary tract infection) and intraoperative findings (laterality of EU and presence of concurrent anatomic anomalies) were descriptively assessed for potential association with outcome. Because of the limited number of cases with post‐operative incontinence (*n* = 3), multivariable analysis and inferential statistical testing were not conducted. Observed trends are presented descriptively.

## 4 Results

### 4.1 Study Population

Nineteen male dogs were diagnosed with intramural EUs and treated using CLA‐EU. One dog was excluded because it was lost to follow‐up before 90 days. As a result, 18 male dogs and 28 ureters (8 unilateral; 20 bilateral) were included. The median age at the time of the procedure was 1.25 years (range, 0.5–8 years). Ten of 18 dogs (56%) were neutered at the time of CLA‐EU. Seven of 8 intact dogs (88%) were neutered after the CLA‐EU procedure. Fourteen of 18 dogs (78%) were reported to be incontinent at the time of presentation, with less common concurrent signs of hematuria (11%) in two dogs, pollakiuria (11%) in two dogs, and stranguria (6%) or polyuria and polydipsia (6%) in one dog each. The median age of onset of urinary incontinence was 6.5 months (range, 1.5 months–7 years). Four of 18 dogs (22%) were continent at the time of diagnosis. Each of these dogs was presented primarily for unrelated clinical signs including vomiting, diarrhea, inappetence, stranguria, pollakiuria, pleural effusion, or some combination of these. These dogs were incidentally diagnosed with EUs during evaluation of their unrelated clinical signs after detection of hydronephrosis and hydroureter on abdominal imaging.

The breeds included 8 Labrador Retrievers, 1 Golden Retriever, 1 Standard Poodle, 1 Newfoundland, 1 Goldendoodle, 1 Entlebucher mountain dog, 1 Bichon Frise, 1 Siberian Husky, and 3 mixed breed dogs. The median body weight at the time of the procedure was 34 kg (range, 6.6–66.4 kg). The median continence score pre‐procedure for the incontinent dogs was 5 out of 10 (range, 1–7.5).

### 4.2 Preoperative Laboratory Data

Pre‐operative CBC and serum biochemistry data were available for all 18 dogs. The CBC was normal in all dogs. The median BUN and serum creatinine concentrations were 17 mg/dL (range, 9–32) and 1 mg/dL (range, 0.7–1.6), respectively. The SDMA data was only available in 12 (67%) dogs, with a median of 13 μg/dL (range, 6–20). Urinalysis and urine microbiological culture were available in all dogs. The median urine specific gravity was 1.038 (range, 1.011–1.050). Four of 18 (22%) dogs had bacterial growth on urine culture within 2 months before the procedure. Two dogs grew more than one bacterium, with one dog positive for *Escherichia coli* and *Pseudomonas aeruginosa* and the other dog positive for methicillin‐resistant coagulase‐negative *Staphylococcus* spp. and *Bacillus* sp. The remaining two dogs grew *E. coli* only. No dog had a reported chronic history of urinary tract infections.

### 4.3 Preoperative Imaging Findings

Seventeen of 18 dogs (94%) had an abdominal ultrasonography performed pre‐procedure by a board‐certified radiologist or an internist, and EU was detected in 7 (41%). One dog (6%) had computed tomography performed that did not identify the EU.

Eleven of 17 dogs (65%) had evidence of unilateral hydronephrosis ipsilateral to the EU (*n* = 8) or bilateral (*n* = 3) hydronephrosis (14 of 28 kidneys, 50%) on preoperative abdominal ultrasound examination. The median size of pelvic dilatation when measured in the transverse plane was 7 mm (range, 1–43). Twelve of 17 dogs (71%) had evidence of unilateral hydroureter ipsilateral to the EU (*n* = 9) or bilateral (*n* = 4) hydroureter (17 of 28 ureters, 61%) on preoperative abdominal ultrasound examination. The median size of ureteral dilatation was 13 mm (range, 2–38) at the widest diameter reported. All four continent dogs with incidentally diagnosed EU had both hydronephrosis and hydroureter on preoperative ultrasonography.

### 4.4 Intraoperative Findings

**FIGURE 1:**
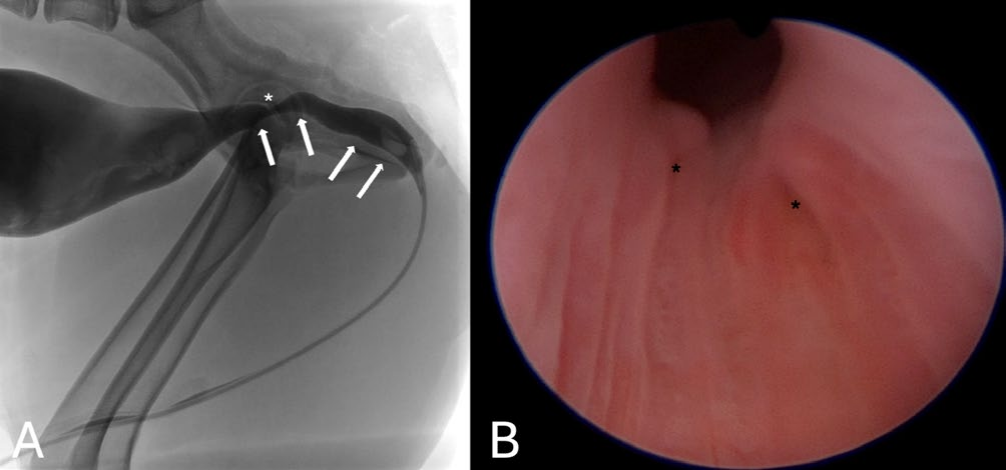
A male dog with bilateral intramural EU. (A) Fluoroscopic contrast image in lateral recumbency showing a dilated urethra (arrows) before and after the colliculus seminalis (white asterisk). (B) Cystoscopic visualization of bilateral intramural ectopic ureters (black asterisks) with evidence of urethral dilation in this region. Abbreviation: EU, ectopic ureter.

Retrograde flexible cystourethroscopy with contrast cystourethrogram and retrograde ureteropyelogram was performed in all 18 dogs to confirm the intramural nature of the EU. Unilateral EU was identified in 8/18 (44%) and bilateral EU in 10/18 (56%), totaling 28 EUs in 18 dogs. Of the eight dogs diagnosed with unilateral EU, three were right‐ and five were left‐sided. All 28 EUs had the orifice located between the caudal aspect of the prostatic urethra and the urethrovesicular junction. Aside from the presence of EU, other anatomical abnormalities included the presence of hydroureter (13/18 [72%] dogs; 17/28 [61%] EUs), hydronephrosis (12/18 [67%] dogs; 14/28 [50%] EUs), EU orifice stenosis (11/18 [61%] dogs; 16/28 [57%] EUs), ureteroceles (7/18 [39%] dogs; 7/28 [25%] EUs) and a dilated urethra (3/18 [17%] of dogs). Hydronephrosis, hydroureter, and EU orifice stenosis were consistently observed in the four continent dogs. A dilated urethra was diagnosed in three dogs based on fluoroscopic and cystoscopic visualization of size in reference to the diameter of the cystoscope and based on the operator's experience (Figure 1).

A 0.025″ guide wire and 4 Fr open‐ended ureteral catheter were used to catheterize all EUs. A 600 μm or 400 μm diode laser was used in 10/18 (56%) and 3/18 (17%) dogs, respectively, and the 600 μm holmium:YAG laser was used in 5/18 (28%). Mean procedure time was 82 min (range, 47–182) with mean time for unilateral CLA‐EU being 78 min and for bilateral being 86 min, including diagnostic retrograde cystourethroscopy, perineal access, and the CLA‐EU procedure. One dog had signs of post‐operative dysuria that resembled functional outflow tract obstruction and resolved after a 5‐day course of tamsulosin and diazepam. No other intra‐ or postoperative complications were noted. Antimicrobial administration consisted of intraoperative IV cefazolin (22 mg/kg) and 3 days of post‐operative PO antibiotics consisting of amoxicillin‐clavulanic acid in 16 dogs, enrofloxacin in 2 dogs, and doxycycline in 1 dog. Post‐operative pain management included 1–3 days of either tramadol or codeine.

## 5 Outcome

The median follow‐up time post‐CLA was 1789 days (range, 98–3560). Seventeen dogs (94%) were alive at the time of the last follow‐up. One dog was euthanized 1397 days post‐CLA‐EU because of the progression of an apical bladder carcinoma.

The median continence score post‐procedure in the 14 incontinent dogs was 10 (range, 1–10; Table S1), without any additional interventions. The preoperative continence scores were significantly different from postoperative continence scores (*p* = 0.001). Eleven of 14 dogs (79%) that were incontinent before CLA‐EU were completely continent with a score of 10 without any additional interventions. The 4 dogs with incidental diagnoses of EU without any history of urinary incontinence remained continent with scores of 10 after CLA‐EU. Overall, 12/14 (86%) dogs were considered continent (scores ≥ 9/10) at the time of last follow‐up. Seven of 8 intact dogs (88%) were neutered after CLA‐EU, which did not impact their continence scores.

Three of 14 dogs (21%) remained incontinent after CLA‐EU, and their pre‐operative and post‐operative continence scores did not change, being 2.5, 2.5, and 1, respectively. One of the three dogs that remained incontinent did not receive any additional interventions. The two other dogs received phenylpropanolamine (PPA; 1.6 mg/kg PO q8h) with methyltestosterone (0.48 mg/kg PO q24h) in one dog and PPA alone (0.77 mg/kg PO q8h) in the other. The first dog showed signs of clinical improvement, with the continence score improving from 2.5 to 9 with the addition of medications. However, after discontinuation of medications, the dog became incontinent again with a score of 2.5, and the owner elected to not pursue long‐term medications. The other dog did not improve with the addition of PPA, and the owners declined methyltestosterone use. This dog received an investigational device (urethral coil) to limit the urethral lumen diameter, and this approach was not successful in improving continence either.

Although several anatomic abnormalities were observed intraoperatively, no statistical analysis of their relationship to outcome could be performed because of the limited number of persistently incontinent dogs. Descriptive statistics for preoperative and intraoperative variables are summarized in Table 2.

**TABLE 2:** Association between multiple variables and final continence score in 18 dogs with EU that had CLA performed.

	Continent (*n* = 15)	Incontinent (*n* = 3)
Age at procedure (mo)‐median (range)	18 (6–132)	12 (6–24)
Weight at time of procedure (kg)‐median (range)	34.2 (7.5–66.4)	18.4 (6.6–30.9)
BUN (mg/dL)‐median (range)	17 (9–32)	24 (15–32)
Creatinine (mg/dL)‐median (range)	1 (0.7–1.5)	1 (0.9–1.3)
SDMA (μg/dL)‐median (range)	12.5 (6–20)	14 (10–18)
USG‐median (range)	1.039 (1.008–1.050)	1.037 (1.028–1.038)
Procedure time (min)‐median (range)	72 (45–182)	75 (60–90)
UTI before procedure	3/15	0/3
Presence of EU orifice stenosis	11/15	0/3
Presence of ureterocele	7/15	0/3
Presence of hydronephrosis	12/15	0/3
Presence of hydroureter	13/15	0/3
Presence of a dilated urethra	0/15	3/3
Bilateral EU	7/15	2/3
Diode laser	11/15	2/3
Already neutered at time of procedure	9/15	1/3
Neutered after procedure	5/15	2/3

Abbreviations: CLA, cystoscopic‐guided laser ablation; EU, ectopic ureter; UTI, urinary tract infection.

Eleven of 18 dogs (61%) had a postoperative abdominal ultrasound examination at least 6 weeks after the procedure that was available for evaluation. The median measurement for renal pelvic dilatation in the transverse plane on the side of the EU treatment was 6.5 mm (range, 2–40 mm), and 3.9 mm (range, 2–20 mm) for ureteral dilatation. These results were associated with improvement from preoperative measurements in 10/14 kidneys for renal pelvic size and 12/17 ureters for ureteral size in the 11 dogs. All four dogs that were already continent before CLA‐EU showed improvement of hydronephrosis or hydroureter or both on follow‐up ultrasonography. Eleven of 18 dogs (61%) had postoperative laboratory test results available for review with a median BUN of 14 mg/dL (range, 4–30) and serum creatinine concentration of 1.3 mg/dL (range, 0.5–1.8), results that were not significantly different from preoperative results (*p* = 0.61 and *p* = 0.55, respectively). Data on postoperative SDMA concentration was not available for most dogs because of the local laboratories used for each dog. No dog was reported to have any chronic or recurrent UTIs after CLA‐EU at the time of last follow‐up at a median of 1789 days (range, 98–3560).

## 6 Discussion

Our results suggest that CLA‐EU is a safe, minimally invasive, and effective procedure to treat intramural EU in male dogs with good to excellent long‐term outcomes. In our study, 18 male dogs underwent CLA‐EU, but four dogs were already continent at presentation. When these dogs were removed from the analysis, 11/14 (79%) of the remaining dogs became completely continent after CLA‐EU without any intervention and 12/14 (86%) after the addition of medications. Additionally, improvement of hydronephrosis and hydroureter was documented in 10/14 kidneys and 12/17 ureters, respectively, after CLA‐EU.

Traditional treatment options for EUs previously have involved surgical techniques such as ureteroneocystostomy, neoureterostomy, and nephroureterectomy [1, 8, 16, 17, 18, 19]. Surgical correction techniques have shown variable success with post‐operative continence rates ranging between 37% and 67% in female dogs [1, 16, 17, 18, 19]. More literature is available on the treatment of EU in female dogs than for male dogs. With the first case series reported in 2012, CLA‐EU has emerged as a safe and effective alternative to traditional surgical management [7]. One subsequent study compared the rate of long‐term (median, 39 months follow‐up) incontinence after intramural EU correction using CLA‐EU versus neoureterostomy and found that the rate of incontinence recurrence after CLA‐EU (0%) was significantly less than that after surgery (42%) in female dogs [20]. Another study reported a novel cystoscopic‐guided scissor transection with continence rates of 43% (3/7) without additional interventions and 86% (6/7) with additional interventions such as the addition of PPA or multiple surgical interventions [21]. Numerous other studies over the past decade have reported successful outcomes with CLA‐EU in female dogs with continence rates ranging between 25% and 72% without additional interventions, and 56%–88% with additional interventions [7, 10, 11, 12, 13].

Pre‐existing veterinary literature evaluating surgical outcomes for management of EUs specifically in male dogs consists of single case reports [22, 23, 24, 25, 26, 27] with only a few larger case series (*n* = 16, 24, 11) exclusively evaluating surgical outcomes [3, 8, 9]. In these studies, post‐operative continence rates without any additional interventions ranged from 74% to 100% [3, 8, 9]. The first and only report of CLA‐EU in 4 male dogs showed that the procedure was safe, effective, and durable with a long‐term postoperative continence rate of 100% at a median follow‐up time of 18 months [6]. Cystoscopic laser ablation of ectopic ureters has been utilized globally and is considered the standard‐of‐care based on the American College of Veterinary Internal Medicine consensus statement for urinary incontinence [28]. Our results coincide with reported outcomes in the veterinary literature in as much as the long‐term continence rate after CLA‐EU of incontinent male dogs in our study was 79% without and 86% with concurrent medications. Additionally, no major complications were observed in this population after CLA‐EU, supporting the premise that this technique could be a safer alternative to avoid the post‐operative complications reported with traditional surgery, including obstruction at the site of reimplantation or neostomy site, ureteral perforation, and bleeding [1, 2, 9, 18]. Future studies are needed to assess additional procedures (e.g., bulking agents, artificial urethral sphincters) and their efficacy in decreasing incontinence in male dogs.

Concurrent urinary tract anatomic abnormalities were found commonly in our population of male dogs, similar to those reported previously [8, 9, 10, 11, 12, 15, 20, 21]. The presence of hydronephrosis (67%) or hydroureter (72%) in our male population was comparable to that reported in females (13%–75% and 14%–75%, respectively). In contrast, evidence of ureteral orifice stenosis (61%) and ureterocele (39%) was higher in males than in females (3%–5% and 3%–18%, respectively) [8, 9, 10, 11, 12, 15, 20, 21]. The presence of ectopic or orthotopic ureteroceles in dogs has been associated with lower urinary tract signs including urinary incontinence, stranguria, and pollakiuria [29, 30, 31, 32]. In our study, all male dogs with concurrent ureteroceles underwent treatment with CLA‐EU, consistent with protocols reported in female dogs [7, 29]. All four continent dogs with incidentally diagnosed EUs exhibited hydronephrosis and hydroureter on preoperative ultrasound examination, with improvement in both on follow‐up imaging. This finding supported the decision to pursue treatment in these dogs, despite the absence of azotemia and lack of glomerular filtration rate studies to assess for potential renal dysfunction. All kidneys and ureters that were re‐evaluated where evidence of hydronephrosis or hydroureter was documented had static or improved ultrasonographic measurements, consistent with prior reports in female dogs [7, 33, 34]. As with female dogs, this dilatation is likely congenital and chronic, and although complete resolution is uncommon, improvement supports the likelihood that the obstructive lesions have been successfully decompressed.

A unique finding in male dogs was that those that remained persistently incontinent (*n* = 3) had a subjectively large proximal urethral diameter noted during cystourethroscopy and cystourethrography. Although this finding suggests a possible association with poor outcome, only a few affected dogs were documented, and more data is needed to draw definitive conclusions. The association between a dilated urethra and urinary incontinence in male dogs is sparsely reported in veterinary literature, but it is also seen in female dogs [7, 28]. The assumption is that failure of urethral mucosal coaptation and a lower urethral closure pressure contribute to the incontinence in both male and female dogs. More studies need to be performed prospectively to definitively correlate urethral diameter, urethral length, and continence rates. Interestingly, all three persistently incontinent dogs in our study had a dilated urethra, yet none of the other dogs that achieved full continence had evidence of this finding, nor did the four male dogs in the prior CLA‐EU study that were fully continent after CLA‐EU [6]. One of the two incontinent dogs that was treated with PPA and methyltestosterone responded well to medical management.

Urinary incontinence is the most common clinical sign in dogs with EUs. Female dogs are reported to be affected 20 times more frequently than male dogs [4], and this observation could be a consequence of the fact that male dogs have longer urethras, with a different distribution of muscle, particularly skeletal muscle, to support their urethral sphincter mechanism [35]. Therefore, male dogs may not display signs of urinary incontinence despite the presence of EU and therefore are not always diagnosed. In our study, 22% of male dogs with EU were continent and their EUs were incidentally diagnosed during abdominal imaging for medical issues unrelated to the EU and were found to have renal or ureteral dilatation. Continence in these dogs also may be a result of ureteral orifice stenosis, because partial obstruction of the outflow tract could have minimized urine leakage. Generally, incontinence in dogs with EU is thought to occur from abnormal drainage of urine distal to the point of maximal urethral closure, and mechanical disturbance of the internal urethral sphincter by the intramural ureter or overall concurrent incompetence of the urethral sphincter mechanism. In urodynamic studies of female dogs with EUs, 89% of dogs had concurrent functional abnormalities of the urethra or bladder [36], in addition to the EU itself. It is hard to determine if male dogs in our study also suffered from concurrent urethral sphincter mechanism incompetence because urethral pressure profiles were not performed. Across multiple studies, urinary incontinence is reported to occur later in life in male dogs compared with females, with some male dogs presenting at up to 4–5 years of age [3, 6, 9, 17, 24]. One study documented that 19% of male dogs with EUs were completely continent [3], and a similar percentage was found in our study (22%). This finding in male dogs is less commonly seen in females, likely because of the proximal origin of the EU within the urethra and the longer urethral sphincter mechanism in male dogs [35]. The presence of EU orifice stenosis and associated hydronephrosis, which was found incidentally on abdominal imaging later in life in these 22% of dogs, supports the idea that concurrent urethral sphincter incompetence is likely less common in male dogs.

A definitive diagnosis of EUs can be made using various imaging modalities such as cystourethroscopy, abdominal ultrasonography, and computed tomography, but sensitivity for detection is reported to be variable [6, 7, 10, 11, 12, 37, 38, 39, 40, 41, 42]. In female dogs, abdominal ultrasonography has a reported sensitivity between 20% and 91% [38, 39, 43], contrast‐enhanced computed tomography 91%–100% [37, 40, 41] and cystourethroscopy 100% [6, 7, 10, 11, 12, 42]. Most of these studies included only female dogs. In our study, only 42% of EUs in male dogs were diagnosed by abdominal ultrasonography and 100% by cystourethroscopic evaluation. Only 1 dog with a left‐sided unilateral EU had computed tomography performed, which did not identify the EU, supporting that in male dogs cystourethroscopy may have the highest likelihood of detecting an abnormally‐located ureteral orifice in male dogs.

Limitations of our study included its retrospective design and reliance on owner‐reported outcomes of pre‐ and postoperative continence. Although our study had a larger sample size compared with the only published study on male dogs with EU treated using CLA‐EU, the total sample size still is relatively low. One strength of our study is that all dogs were managed by the same clinicians pre‐ and post‐operatively, and the same operator performed all CLA‐EU procedures, and thus consistency in treatment in the short‐ and long‐term was high. Another limitation to our retrospective study was defining urethral diameter. Imaging from video urethroscopy and cystourethrography was used to confirm the visibly large urethral diameter compared to a typical male dog's urethral size at the time of procedure. However, this assessment was not compared to anatomically normal dogs using a measurement tool. The diagnosis of a dilated urethral lumen was subjective based on the authors' extensive experience.

In conclusion, treatment of EUs in male dogs using CLA‐EU had positive short‐ and long‐term outcomes with complete resolution of urinary incontinence in 79% of incontinent male dogs without any intervention, and in 86% with the addition of medications. All dogs that were persistently incontinent had a subjectively dilated proximal urethra visualized, and this finding is likely a factor that can negatively impact postoperative success, although one dog did respond positively to medical management.

## Disclosure

Authors declare no off‐label use of antimicrobials.

## Ethics Statement

Authors declare no institutional animal care and use committee or other approval was needed. Authors declare human ethics approval was not needed.

## Conflicts of Interest

The authors declare no conflicts of interest.

## Supporting information


**Table S1:** Continence score Pre versus Post CLA‐EU. Abbreviations: CLA, cystoscopic‐guided laser ablation; EU, ectopic ureter; U‐Coil, urethral coil.
