# Supplementary material for: Cystoscopic‐Guided Laser Ablation of Intramural Ectopic Ureters in Male Dogs: A Retrospective Study of 18 Cases (2011–2023)
Source: J Vet Intern Med. 2025 Sep 10;39(5):e70243. doi: 10.1111/jvim.70243 (PMC12421311; doi:10.1111/jvim.70243)
Supplement: Supplementary file 1 — Table S1: Continence score Pre versus Post CLA‐EU. Abbreviations: CLA, cystoscopic‐guided laser ablation; EU, ectopic ureter; U‐Coil, urethral coil. [file JVIM-39-e70243-s001.docx]

| Male dogs | Continence score pre CLA-EU | Continence score post CLA-EU without any intervention | Continence score with addition of meds | Continence score with additional U-Coil procedure |
| --- | --- | --- | --- | --- |
| 1 | 5 | 10 |  |  |
| 2 | 5 | 10 |  |  |
| 3 | 10 | 10 |  |  |
| 4 | 1 | 10 |  |  |
| 5 | 10 | 10 |  |  |
| 6 | 2.5 | 2.5 | 9 |  |
| 7 | 10 | 10 |  |  |
| 8 | 1 | 10 |  |  |
| 9 | 2.5 | 2.5 | 2.5 | 2.5 |
| 10 | 7.5 | 10 |  |  |
| 11 | 5 | 10 |  |  |
| 12 | 2.5 | 10 |  |  |
| 13 | 10 | 10 |  |  |
| 14 | 5 | 10 |  |  |
| 15 | 5 | 10 |  |  |
| 16 | 5 | 10 |  |  |
| 17 | 1 | 1 |  |  |
| 18 | 7.5 | 10 |  |  |

**Supplemental Table 1**: Continence score Pre vs Post CLA-EU. Abbreviations: CLA; cystoscopic-guided laser ablation, EU; ectopic ureter, U-Coil; urethral coil.
